# Camera-in-the-Loop Realization of Direct Search with Random Trajectory Method for Binary-Phase Computer-Generated Hologram Optimization

**DOI:** 10.3390/jimaging11120434

**Published:** 2025-12-05

**Authors:** Evgenii Yu. Zlokazov, Rostislav S. Starikov, Pavel A. Cheremkhin, Timur Z. Minikhanov

**Affiliations:** Laser Physics Department, Institute for Laser and Plasma Technologies, National Research Nuclear University MEPhI (Moscow Engineering Physics Institute), Kashirskoe Shosse 31, 115409 Moscow, Russia

**Keywords:** computer holography, computer-generated hologram, aberration correction, holographic display, spatial light modulator, camera-in-the-loop method

## Abstract

High-speed realization of computer-generated holograms (CGHs) is a crucial problem in the field of modern 3D visualization and optical image processing system development. Binary CGHs can be realized using high-resolution, high-speed spatial light modulators such as ferroelectric liquid crystals on silicon devices or digital micro-mirror devices providing the high throughput of optoelectronic systems. However, the quality of holographic images restored by binary CGHs often suffers from distortions, background noise, and speckle noise caused by the limitations and imperfections of optical system components. The present manuscript introduces a method based on the optimization of CGH models directly in the optical system with a camera-in-the-loop configuration using effective direct search with a random trajectory algorithm. The method was experimentally verified. The results demonstrate a significant enhancement in the quality of the holographic images optically restored by binary-phase CGH models optimized through this method compared to purely digitally generated models.

## 1. Introduction

Computer holography [1] is a technology that is in high demand in a variety of applications, including 3D visualization [2], augmented reality displays [3], holographic data storage [4], security printing [5,6], optical computing [7], etc. The development of high-resolution spatial light modulators (SLMs) has garnered interest in computer holography methods, leading to the introduction of an approach known as electronic holography [8]. Models of modern SLMs with a resolution of several megapixels and a refresh rate of more than 1 kHz include those based on the use of ferroelectric liquid crystals on silicon (FLCOS) or digital micro-mirror device (DMD) technologies. However, these devices are limited in terms of their light modulation capabilities, providing only a binary type of modulation, such as phase-only modulation in the case of FLCOS and amplitude-only modulation in the case of DMDs [9]. Furthermore, the quantization of computer-generated hologram (CGH) models down to two levels (binarization) and the imperfections of optical system components lead to the appearance of background noise and significant distortions in the holographic images produced.

The main approaches of modern computer holography include the following: (1) direct calculation of CGH fringe patterns using integral transforms of scalar diffraction theory; (2) kinoform generation via the optimization of object beam spatial-phase portraits; and (3) neural holography methods, which use trained image generation models for fringe pattern synthesis.The application of the first of these methods, such as in determining bipolar intensity [10], double-phase coding [11,12,13], Lee–Buckhard vector coding [14,15], or binary superpixel-based complex modulation [16], allows us to obtain fringe patterns capable of restoring images with a given amplitude and phase distribution, which is especially important for the restoration of phase-modulated complex data pages in holographic data storage systems [17,18] or the realization of complex spatial Fourier filters in coherent diffractive correlators [19]. However, the major drawback of these methods is their limited field of view due to the presence of unwanted diffraction orders and their instability under quantization; these drawbacks limit their use in applications for visual perception.

Kinoform generation methods are especially attractive in 3D visualization problems due to the high quality of holographic images that can be obtained without additional diffraction orders. These methods, including the Grechberg–Saxton algorithm (GSA) [20], direct search methods [21,22], the stochastic gradient decent (SGD) method [23], and so on, are mainly based on the iterative calculation of light propagation integrals and the tuning of the CGH fringe pattern model in order to enhancement reconstruction quality. Recently, the two-stages method for synthesizing and optimizing the binary CGH model was introduced [24]. It was shown that holographic images restored by binary CGH generated using GSA can be sufficiently improved by means of normalized standard deviation (NSTD) and diffraction efficiency (DE) with the use of direct search with random trajectory (DSRT) method.

Although the kinoform-restored holographic images can be of high quality, the long computation time due to the iterative evaluation of light propagation integrals is a significant factor limiting its use in modern applications. Neural holography methods [25] allow for the generation of holographic fringe patterns, including binary CGHs [26] with high generation speed. However, the quality of the reconstructed images depends on the quality of the ground truth dataset used during training in the case of supervised learning [26], or the accurate representation of the optical system model, in the case of unsupervised learning [27]. This means that high-quality fringe patterns generated by traditional methods are in demand at the training stage.

The common problem for most computer holography techniques is the representation of a real optical system in CGH synthesis algorithms. While the calculation algorithms rely on an idealized system model, the real optical system, with its unique imperfections and misalignment, introduces distortions into the read-out beam, resulting in the degradation of the restored image quality. Measuring aberrations can help to improve the quality of restored holographic images, but it does not suppress speckle noise efficiently. Speckle noise is the main factor limiting the perceptual quality of the optical reconstruction of holographic images, especially in the case of the application binary CGHs [28]. Various approaches have been proposed to reduce speckle noise, including mechanical dispecklers [29] and phase masks [30], electrooptic dispecklers [31], and temporal and spatial CGH multiplexing methods [32,33,34,35,36]. While schemes based on additional mechanical or electro-optic components can be bulky, temporal multiplexing methods based on the averaging of several frames limit application of time-division methods for multicolor and 3D holographic image restoration [37,38,39].

The camera-in-the-loop (CITL) approach relies upon the optimization of the CGH fringe pattern directly in the optical system, which is intended to be used for the restoration of holographic images [40,41,42]. CITL basically relies on the iterative registration of restored holographic images via a CMOS camera, calculating the error and evaluating the stochastic gradient decent (SGD) algorithms that generate an updated matrix for the CGH model displayed. Since the CITL approach aims to optimize the CGH fringe pattern in the optical system in situ, the quality of the optical reconstruction by a single CGH is expected to be high, with suppressed speckle noise and distortions. The problem with SGD algorithms is their reliance on a numerical model of the optical system for error back-propagation calculations.

Implementing the DSRT method for binary CGH model optimization directly in the optical system using the CITL approach is attractive for the following reasons: (1) it allows us to obtain CGH models optimized for a given optical system, suppressing distortions and speckle noise; (2) it accelerates computations compared to a numerical implementation of the DSRT algorithm, since restored holographic images can be obtained optically and no light propagation integrals need to be calculated; (3) it does not require the use of a real system model in the calculation algorithms. This paper presents the study results of the experimental implementation of the DSRT method by means of the CITL approach for binary-phase CGH optimization. The paper is organized as follows: The next section presents the basic structure of the binary CGH synthesis algorithm, which includes a GSA block, followed by an optimization block, based on the DSRT method. Section 3 focuses on the experimental investigation of the possibilities and features of the DSRT method’s optical implementation. It includes a description of an experimental optical setup based on a liquid crystal on silicon (LCOS) SLM and a CMOS camera, presents the results of binary-phase CGH synthesis using a three-stage algorithm based on both numeric and optical implementations of the DSRT method, and compares the three-stage and two-stage algorithms. The latter case is based only on the optical implementation of the DSRT method. Section 4 discusses the computational burden of numeric and optical implementations of DSRT and the possibilities of the DSRT method’s optical implementation in schemes based on state-of-art high-speed binary SLMs. Section 5 summarizes the main study results.

## 2. Direct Search with a Random Trajectory Method

**Figure 1:**
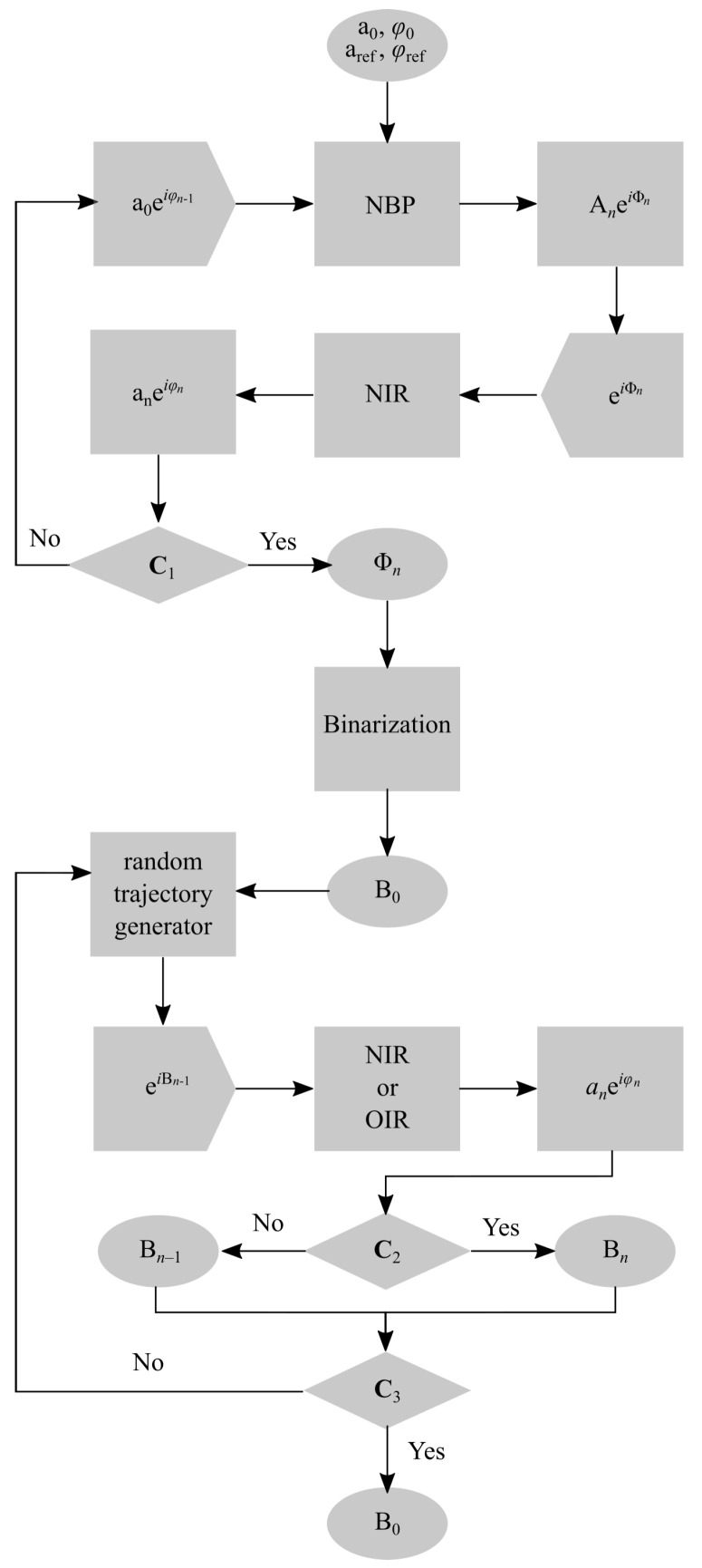
Operational flowchart of CGH synthesis and optimization algorithm.

Figure 1 shows the operational flowchart of the implemented two-stage CGH synthesis algorithm. The first stage realizes a standard iterative GSA with the following input parameters: $a_{0}$—the matrix of the object beam spatial amplitude distribution that represents the holographic image; $\phi_{0}$—the matrix of the initial object beam phase factor to be optimized; $a_{\mathrm{ref}}$ and $\varphi_{\mathrm{ref}}$—the matrices of amplitude and phase distributions of the reference beam. Starting from the initial distribution matrix of the holographic image complex amplitude $u_{0}=a_{0}\exp(i\phi_{0})$, the algorithm iterates through the computation of numerical backward propagation (NBP) onto the hologram plane, hologram amplitude unification, and numerical image reconstruction (NIR) until the conditional test **C**_1_ is satisfied. NBP includes the calculation of the reversed propagation integral and the division of the result on the reference beam complex amplitude function. NIR includes multiplication of the phase-only function of kinoform on the reference beam complex amplitude function and the calculation of the forward propagation integral. If on *n*-th iteration condition **C**_1_ is satisfied, the iterations stop, and the algorithm produces the phase distribution function $\Phi_{n}$ for binarization and further processing. Condition **C**_1_ calculates and analyzes the error function $L_{n}$ [43]. Similar to [24], the following loss function was used within this work:

(1) $$L_{n}=\alpha \sigma_{n}+\left(1-\alpha \right)\left(1-\eta_{n}\right),$$

where α∈[0,1] is a tunable parameter,

(2) $$\sigma_{n}=\sqrt{1-\frac{{\left(\sum_{x,y=1}^{X,Y}a_{0}\left[x,y\right]a_{n}\left[x,y\right]\right)}^{2}}{\left(\sum_{x,y=1}^{X,Y}a_{0}^{2}\left[x,y\right]\right)\left(\sum_{x,y=1}^{X,Y}a_{n}^{2}\left[x,y\right]\right)}},$$

is NSTD, and

(3) $$\eta_{n}=\left(\sum_{\left(x,y\right)\in \Omega_{a}}I_{n}\left[x,y\right]\right)/\left(\sum_{\left(x,y\right)\in \Omega_{h}}I_{n}\left[x,y\right]\right),$$

is DE. $a_{0}$ and $a_{n}$ are the matrices of the ground truth image and the holographic image, restored on *n*-th iteration, respectively. X,Y denote the image sizes in pixels, $I_{n}$ is the CGH diffraction field restored on the *n*-th iteration, $\Omega_{a}$ is the holographic image area in a + 1 diffraction order, and $\Omega_{h}$ is the CGH diffraction field area within the CGH Nyquist aperture, $\Omega_{a}\in \Omega_{h}$. The stagnation of $L_{n}$ can be used as the truncation condition, as was done in [24], or the number of iterations can be manually limited.

The generation of a CGH fringe pattern model using GSA allows for the optimization of the initial random phase distribution of the object beam into a specific distribution of the spatial phase portrait that provides the increased quality of the holographic image reconstruction from an amplitude-only or phase-only kinoform. However, stagnation of the GSA after several dozen iterations results in limited quality improvement. As previously shown [24], a sufficient increase in reconstruction quality can be achieved if the binary GSA-generated fringe pattern is subjected to the DSRT method. Thus, the next stage of the algorithm is the implementation of the DSRT method.

At the input of the second stage, the GSA-generated fringe pattern $\Phi_{n}$ is sent to the binarization module, which converts the smooth function $\Phi_{n}$ into a distribution of 0 and π values, labeled as $B_{0}$ in Figure 1. Various binarization techniques were compared for implementation in the case of the application of the DSTR to optimize the CGHs for realization using DMD [28]. It was shown that the Otsu global threshold method may be suboptimal for binary amplitude-only modulation. In the given paper, the generation of binary-phase CGHs is under study. Due to the the lack of similar studies dedicated to binary phase-only holograms, the Otsu global threshold method was selected for use in this paper. After binarization, a random trajectory (RT) for passing through the CGH pixels is generated. RT is the order governing the selection of $B_{0}$ pixels to be switched at each iteration step of the DSRT algorithm, which includes following procedures: (1) the switch of the CGH model selected pixel (0 to π and vice versa); (2) holographic image restoration; and (3) the loss function calculation. If the gradient of the loss function $\Delta L_{n}=L_{n}-L_{n-1}$ satisfies condition **C**_2_, the new binary pixel value is stored in memory; otherwise, the previous value is retained. Since holographic image restoration can be performed both numerically (NIR) and optically (OIR), two implementations of the DSRT algorithm will be considered below: numeric (nDSRT) and optical (oDSRT). The truncation condition **C**_3_, which simply limits the number of iterations, is used in this paper.

## 3. Experimental Verification of the Method

This section describes the optical setup used for holographic image reconstruction and DSRT method realization. Further, the results of two cases of CGH synthesis algorithm implementation in the setup are presented. We examined two different CGH synthesis algorithm structures. The first is a three-stage algorithm that contains consequent GSA, nDSRT, and DSRT blocks. The second is a two-stage algorithm in which the DSRT method is optically implemented directly after the numerical GSA block.

### 3.1. Optical Setup

**Figure 2:**
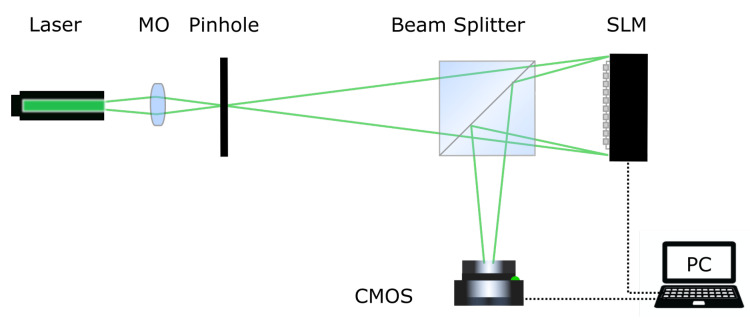
Experimental setup.

Figure 2 represents the experimental setup for the optical implementation of the DSRT method. A Cobolt Samba Nd:YAG laser with a wavelength of 532 nm and an output power of 200 mW was used as the light source. A microlens and a pinhole diaphragm formed a divergent spherical reference beam. A HoloEye PLUTO-2-VIS-016 LCOS SLM with a resolution of 1920×1080 and a pixel pitch of 8 μm was mounted 90 cm from the pinhole diaphragm. A Flare 48M CoaXPress CMOS camera with a resolution of 7920×6004, a full resolution frame rate 30.9 fps, and a pixel pitch of 4.6 μm was used to acquire the restored holographic images. Since the LCOS SLM is a reflective display, a beam splitter cube was used in the setup. The optical distance between the SLM and the camera was 45 cm. The SLM and the camera were synchronized with a 60 Hz signal, but both were programmed to perform the iterative display of CGHs and to acquire the holographic image at a frame rate of 5 Hz. The camera exposure time was set to 8.5 ms. This limitation were due to the low response time of the LCOS SLMs, as measured previously [44,45]. Both the SLM and CMOS camera were connected to a personal computer, which implemented the CGH synthesis and realization algorithms, as well as the acquisition, digitization, and processing of the restored holographic images.

Since the distance between the pin-hole diaphragm and the SLM was 90 cm, and the distance between the SLM and the camera was 45 cm, the equivalent focusing distance of the CGH was $z_{0}=30$ cm. This allows for the use of a Fresnel integral calculation algorithm based on a single discrete fast Fourier transformation to compute the forward and backward propagation of the reconstructed beam when implementing NBP or NIR procedures. According to this method, the complex matrix of the reference beam in image plane a can be determined from the complex matrix of the reference beam in hologram plane A using the following equation:

(4) $$a=h\circ F\left[A\circ H\right],$$

where F[] is the two-dimensional discrete Fourier transformation operator, ∘ is the Hadamard product, and h and H are spatial phase chirp matrices, which can be presented as follows:

(5) $$h\left[x,y\right]=\exp\left(j\pi \left(\frac{x^{2}}{\mu_{x}X}+\frac{y^{2}}{\mu_{y}Y}\right)\right),$$

(6) $$H\left[x,y\right]=\exp\left(j\pi \left(\frac{\mu_{x}x^{2}}{X}+\frac{\mu_{y}y^{2}}{Y}\right)\right),$$

where $\mu_{x}$ and $\mu_{y}$ are scaling factors along horizontal and vertical directions, which can be defined by the parameters of the optical system $z_{0}$, the λ and SLM pixel pitches $\Delta_{x}$ and $\Delta_{y}$, and CGH resolutions *X* and *Y*, such as

(7) $$\mu_{k}=\frac{K\Delta_{k}^{2}}{\lambda z_{0}},$$

where k=x|y and K=X|Y for horizontal and vertical directions, respectively. Since h and H are independent from object function, these matrices can be stored in memory and used at both the GSA and nDSRT stages.

The experiments used CGH models with a resolution of 128×128 pixels. Each model was scaled by a factor of 8 before realization in the optical setup in order to fill the SLM aperture. Using the parameters of the optical setup, Equation (7), and considering a scaling factor of 2 due to the divergence in the read-out beam [24], the physical dimensions of the Nyquist aperture of the optical system can be calculated at about 5×5 mm, corresponding to the 1084×1084 pixels of the CMOS camera sensor.

### 3.2. Three-Stages Algorithm Implementation

**Figure 3:**
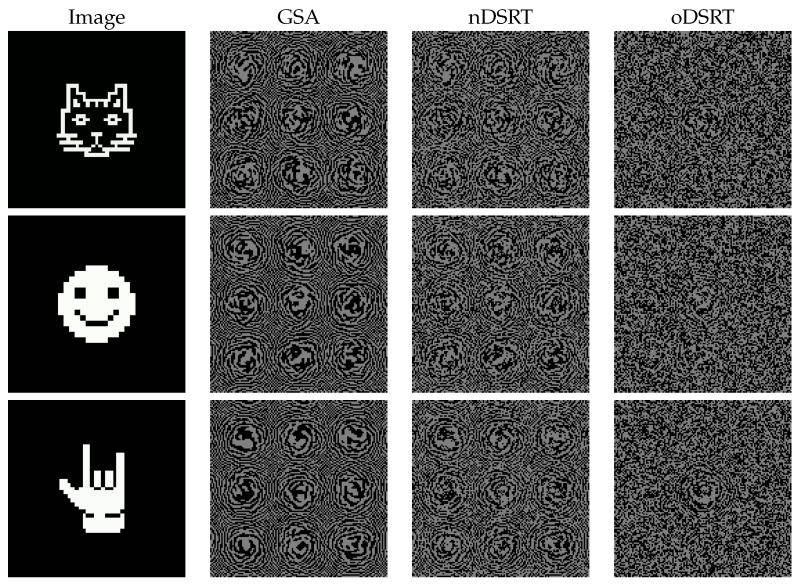
Object images “cat”, “smile”, and “rock” and CGH model fringe patterns obtained at different stages of the three-stage algorithm’s implementation.

Since most commercially available SLMs have equal pixel pitch along the horizontal and vertical directions ($\Delta_{x}=\Delta_{y}$), a square matrix should be used to represent the CGH model in order to avoid the deformation of holographic images due to differences in scale factors. Figure 3 represents the 64×64 objects centered in a 128×128 object field that were used in the experiments, as well as the corresponding 128×128 binary CGH models that were obtained at the end of each stage of the three-stage algorithm’s implementation.

For the purposes of this study, the number of iterations for the GSA stage was limited to 200 (condition **C**_1_), the number of iterations for the nDSRT was limited to 15 cycles of the full 128×128 elements update, and the number of iterations for oDSRT was set to 4 cycles of the full 128×128 elements update. Parameter α (Equation (1)) was set to 0.5 during the nDSRT stage. This selection allows for the equal contribution of both NSTD and DE to the target function evolution, as shown in [24]. In the case of oDSRT. α was set to 0.9 since the optically restored images obtained after the realization of nDSRT-optimized CGHs appeared strongly distorted by speckle noise. The emphasis on NSTD during the oDSRT stage helped us to improve the quality of the restored holographic images by suppressing speckle and background noises. See also Section 3.4 on the way the coefficient α selection affects the quality of CGH optimization in the oDSRT stage.

**Figure 4:**
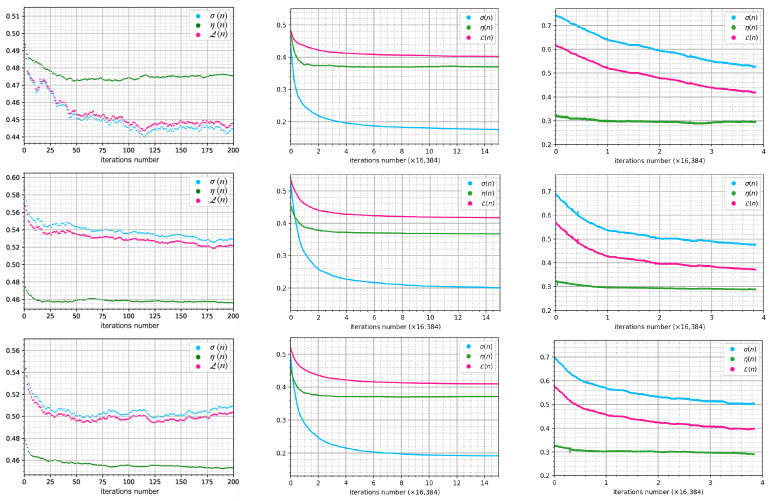
σ(n), η(n), and L(n) in accordance with the iteration number *n* for the GSA (**left column**), nDSRT (**center column**), and oDSRT (**right column**) stages of the three-stage algorithm implementation for the objects “cat” (**top row**), “smile” (**middle row**), and “rock” (**bottom row**).

Figure 4 represents the dependencies of σ(n), η(n), and L(n) on the number of iterations observed in the three stages of CGH generation and optimization. It can be seen that in the GSA stage, stagnation of the target function was achieved after about 50 iterations. The implementation of nDSRT for the optimization of the GSA-generated binary-phase CGH model provides significant improvement in the quality of the numerical reconstruction of the holographic image, reaching 99% of improvement within two–six cycles of a full update of the 128×128 matrix according to a random trajectory. Stagnation of the oDSRT stage was observed after three cycles of the full update of the 128×128 matrix, showing 29–32% improvement via NSTD in all three cases.

**Figure 5:**
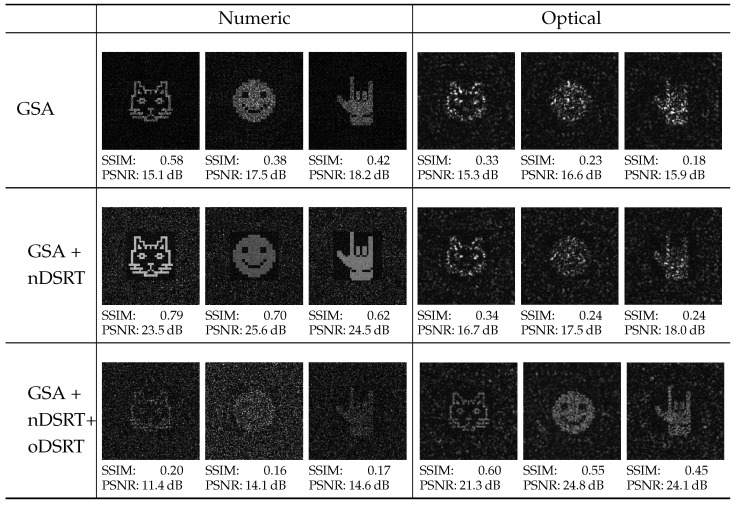
Holographic image reconstructions obtained during the experimental implementation of the three-stage method.

Figure 5 represents the results of the numerical and optical holographic images reconstructed by CGHs, obtained at different stages of the algorith’s implementation, as well as the results of the numerical analysis of these images using the structured similarity index measure (SSIM) and the peak signal-to-noise ratio (PSNR).

Figure 4 and Figure 5 show that GSA-generated holograms provide up to 0.45–0.47 of the DE of numerically restored images, with sufficient quality to distinguish object details. The following nDSRT stage presents a significant improvement of the numerically restored holographic images by means of NSTD (minimum 0.19), SSIM (maximum 0.79), and PSNR (maximum 25.6 dB), with a reduction in DE of 15%. The physical realization of both binarized GSA-generated CGHs and nDSRT-optimized binary-phase CGHs in the optical system using the phase-only SLM showed a significant degradation in the quality of the optically restored holographic images that appeared distorted by speckle noise with almost invisible details. Nevertheless, both the SSIM and PSNR values demonstrate slight improvement in the optical reconstruction quality after CGHs optimization in the nDSRT stage for all three objects. For the optical reconstruction of holographic images by the nDSRT-optimized CGHs, the minimum NSTD obtained is 0.68, which is consistent with value obtained for the optical reconstruction of GSA-synthesized CGH. The maximum SSIM value is 0.34, and the maximum PSNR is 18.0 dB. The implementation of the oDSRT stage resulted in a sufficient improvement in the quality of the optically restored holographic images, providing detailed reconstruction with suppressed speckle noise and low background level. For oDSRT-optimized CGHs, the minimum NSTD value obtained is 0.46, the maximum SSIM value is 0.60, and the maximum PSNR value is 24.8 dB. The measured diffraction efficiency for GSA-generated CGH in the optical setup ranged from 0.37 to 0.39; for nDSRT-optimized CGHs, it was about 0.32, and for oDSRT, it was about 0.28 for all three cases. During the physical realization of GSA-synthesized and binarized CGHs and nDSRT-optimized binary-phase CGHs in the optical setup, a 25% reduction in DE was observed compared to the numerical results. This reduction may be due to imperfections in the optical setup that were not considered in the numerical simulations, such as the non-uniform spatial distribution of read-out beam intensity, aberrations, the non-uniform spatial phase shift of the LCOS SLM, and reflections from the SLM pixels not involved in the CGH display.

Interesting results were obtained following numerical reconstruction of the holographic images using oDSRT-optimized CGHs. The quality of the images was significantly lower than that of the images numerically restored by GSA-generated and nDSRT-optimized CGHs. This indicates the sufficient difference between the ideal system model used in the numerical simulations and the real optical system.

### 3.3. Two-Stage Algorithm Implementation

The two-stage algorithm was experimentally investigated to determine whether adding a nDSRT stage between the numerical GSA and oDSRT stages could provide better results. The CGH models of the “smile” and “rock” objects obtained after the GSA stage (see Figure 3) were selected for optimization by the oDSRT algorithm in the experiments implementing the two-stage algorithm. The coefficient α in these experiments was set to 0.9.

**Figure 6:**
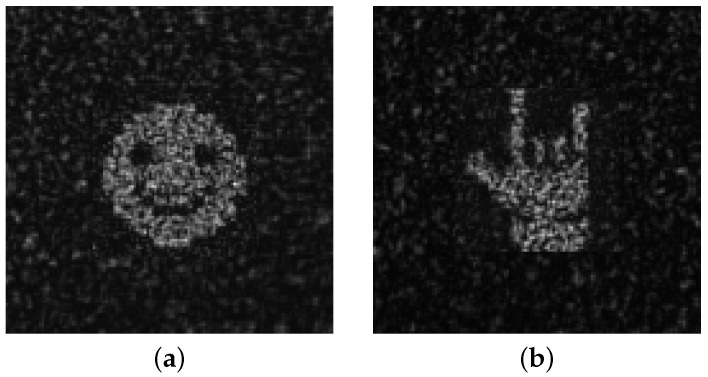
Holographic images of “smile” (**a**) and “rock” (**b**) restored by CGHs optimized via the oDSRT method during the implementation of the two-stage algorithm.

Figure 6 shows holographic images acquired in the experimental setup during the realization of binary-phase CGHs that were synthesized by the two-stage algorithm. Table 1 presents the obtained NSTD, DE, SSIM, and PSNR values. A comparison of these results with those of the three-stage algorithm reveals a 2–4% reduction in reconstruction quality by means of NSTD, SSIM, and PSNR for the two-stage algorithm. However, the DE values obtained by the two-stage algorithm are 19% higher then those fobtained by the three-stage algorithm.

### 3.4. Parameter α Selection in the oDSRT Stage

**Figure 7:**
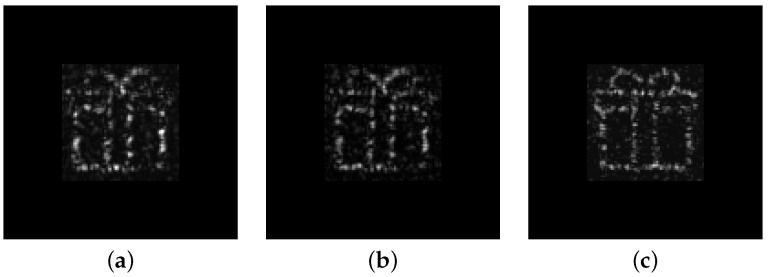
Holographic images optically restored by (**a**) GSA-generated CGH; (**b**) oDSRT-optimized CGH with α=0.6; (**c**) oDSRT-optimized CGH with α=0.9.

The correct selection of coefficient α in the oDSRT stage is important for the DSRT convergence rate and the final quality of the optimized CGH. To study the effect of coefficient α selection, two cases were implemented in the experimental setup: α=0.6 and α=0.9. A new object, “present”, was used in the experiment. CGH generated by the GSA algorithm was used as the starting point for the oDSRT stage. For our purposes, only one full CGH update cycle of 128×128 pixels was performed in both cases. Figure 7 depicts the images restored by CGHs in the optical setup during these experiments.

**Figure 8:**
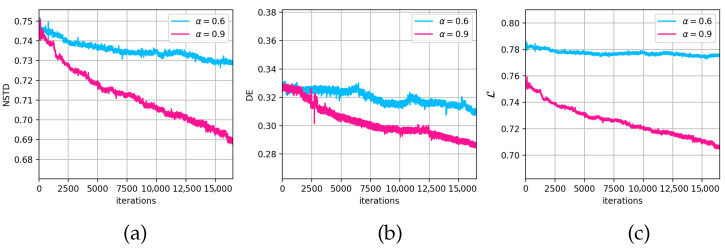
Comparison of NSTD (**a**), DE (**b**), and target function (**c**) evolution for cases of α equal to 0.6 and 0.9.

Figure 8 illustrates the comparison of the NSTD, DE, and target functions evolution depending on the iteration step number during a single all-pixel update cycle. It is clear that although there is no significant difference in the DE evolution for both cases, the NSTD and target function differ significantly. Table 2 shows the result of the main metrics analysis for GSA-synthesized CGH and oDSRT-optimized CGH with α=0.6 and α=0.9.

The results demonstrate that selection of lower values of α leads to the slower decay of the target function. In the case of α=0.6, the target function decreased to only about 1% during a single all-pixel update cycle, whereas for α=0.9, it changed to about 7%.

## 4. Discussion

The experimental results demonstrate that if DE is less sufficient than NSTD, the computationally greedy nDSRT stage does not provide an effective contribution to improving restored holographic images. The diagrams in Figure 4 show that the nDSRT algorithm required about five–six cycles of a full matrix of 128×128 elements, updated according to the selected random trajectory, until it reached stagnation of the target function. The following equation can be used to estimate the complexity of the nDSRT algorithm in a single iteration:

(8) $$N=\left(XY\right)\log_{2}\left(XY\right)+2\left(XY\right)+4\left(XY\right)$$

where X×Y is the CGH model resolution. The first and the second terms result from the calculation of the fast Fourier transformation (FFT) algorithm according to Equation (4). The third term of Equation (8) corresponds to the number of matrix multiplications required to calculate the NSTD for restored holographic images. No matrix multiplications are used when calculating the DE. When running the nDSRT algorithm on a typical desktop PC, processing for a 128×128 model takes about 4 min. According to Equation (8), the optimization time for the $10^{6}$ pixel CGH model would require more than 6 h of computation.

With the implementation of oDSRT, holographic images are obtained optically; no calculations based on the FFT algorithm are required. The first term of Equation (8) can be neglected. The number of multiplications per single iteration becomes proportional to the CGH resolution, which can be performed within 1 ms using modern desktop PCs for matrices with more than $10^{6}$ elements. The main factor that determines the processing time for the optical implementation of the DSRT algorithm is the frame rate of the SLM used in the scheme for CGH displaying. In the experiments, a LCOS SLM was used in the optical setup. As noted, the frame rate of SLM was set to 5 Hz due to the long response time of liquid crystal cells. The plots on the right in Figure 4 demonstrate that oDSRT stagnates after three–four full pixel update cycles. The total processing time for a 128×128 pixel CGH model via the oDSTR method was about 3.5 h.

**Figure 9:**
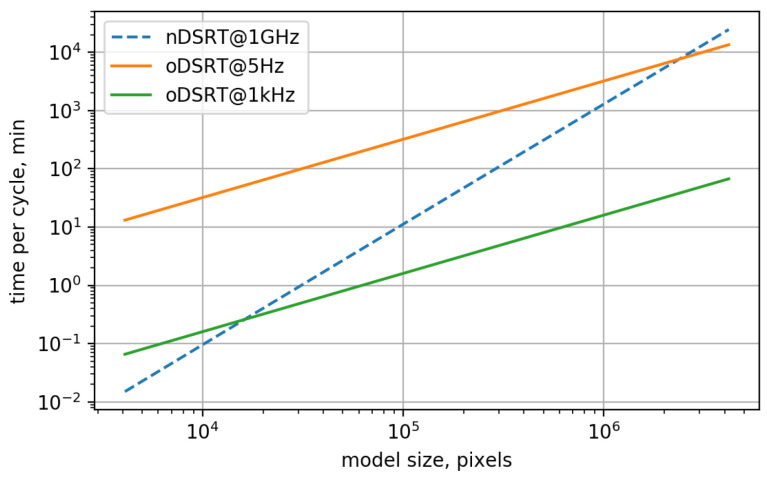
Time per cycle of full matrix update in accordance with model size for implementations of nDSRT with a processor frequency of 1 GHz and oDSRT at an SLM frame rate of 5 Hz and 1 kHz.

Figure 9 shows the estimated time values per full matrix update cycle in accordance with matrix size for the numerical and optical implementations of the DSRT algorithm. For simplicity, the processor frequency in the case of nDSRT was set to 1 GHz. It can be seen that for the CGH models presented by matrices with $10^{6}$ elements, the processing time of the nDSRT algorithm becomes similar to the processing time of the oDSRT algorithm, with a 5 Hz refresh rate, which is about 100 h. However, considering the 1 kHz refresh rate supported by modern FLCOS SLMs, the optical implementation of the DSRT algorithm for a CGH model with $10^{6}$ pixels becomes two orders of magnitude faster than the numerical implementation.

The application of the oDSRT method to optimize CGH with $10^{6}$ pixels and more at a frame rate above 1 kHz requires the high-speed acquisition and processing of restored holographic images. The camera used in the optical setup described in Section 3.1 is a scientific camera capable of capturing 128×128 images, with a maximum frame-rate of about 1093 fps in region-of-interest (ROI) mode. However, the real resolution of the images that must be obtained in the scheme, limited by the Nyquist aperture defined by a CGH pixel size of 36 μm, was about 1084×1084 pixels. The maximum frame rate of the camera in this case can be about 175 fps. However, modern cameras can register 1024×1024 images at a frame-rate of about 1000 fps. The application of the optical setup described in Section 3.1 to optimize CGH models with a resolution of 1024×1024 pixels would require the use of an 8674×8674 pixel CMOS sensor with a pixel pitch of 4.6 μm, which is beyond the available resolution of the camera. This problem can be solved by using a camera or SLM with a higher pixel pitch or by downscaling the restored images using optical projection.

## 5. Conclusions

Based on the results presented in the article, the following conclusions can be drawn:

1. CITL implementation of the DSRT method enabled quality improvement in the restored holographic images of up to 32% by means of NSTD, up to 200% by means of SSIM, and up to 8 dB by means of PSNR, compared to purely numerically synthesized and optimized binary-phase CGH models. In the numerical stages of the proposed algorithms, an idealized model of the optical system was used, ignoring such optical system imperfections as aberrations and readout beam non-uniformity.
2. Using a three-stage CGH synthesis algorithm, including the numerical implementation of GSA, followed by numerical and optical implementations of the DSRT method, produced CGH models with a diffraction efficiency of up to 19% higher than that of models synthesized using a two-stage algorithm, which included only the numerical implementation of GSA and the optical implementation of DSRT. However, the improvements in reconstruction quality by means of the NSTD, SSIM, and PSNR were insufficient, amounting to only 2–4%.
3. Selecting the target function parameter α closer to 1.0 during the implementation of the oDSRT stage leads to a faster decay of the target function value.
4. Implementing the DSRT method by means of the CITL approach for CGH models with a resolution of up to $10^{6}$ pixels using modern high-resolution binary-phase modulators, such as the FLCOS SLMs, has the potential to reduce the computational load by up to two orders of magnitude compared to the purely numerical implementation of the DSRT method. However, this requires a high-speed camera with a larger pixel pitch.

## Figures and Tables

**Table 1:** Quality metrics obtained for images restored by CGHs synthesized by the two-stage algorithm.

Object	NSTD	DE	SSIM	PSNR [dB]
“smile”	0.47	0.37	0.53	24.1
“rock”	0.54	0.34	0.44	22.9

**Table 2:** Quality metrics obtained for GSA-synthesized CGH and oDSRT-optimized CGH with α=0.6 and α=0.9.

	NSTD	DE	SSIM	PSNR [dB]
GSA	0.75	0.33	0.51	25.7
oDSRT (α=0.6)	0.73	0.31	0.57	27.2
oDSRT (α=0.9)	0.69	0.29	0.61	28.1

## Data Availability

The raw data supporting the conclusions of this article will be made available by the authors on request.

## Abbreviations

The following abbreviations are used in this manuscript:

CGH	Computer-generated hologram
SLM	Spatial light modulator
LCOS	Liquid crystal on silicon
FLCOS	Ferroelectric liquid crystal on silicon
DMD	Digital micro-mirror device
GSA	Gerchberg–Saxton algorithm
DSRT	Direct search with random trajectory
NSTD	Normalized standard deviation
DE	Diffraction efficiency
CITL	Camera-in-the-loop
NBP	Numeric backward propagation
NIR	Numeric image reconstruction
FFT	Fast Fourier transformation
PC	Personal computer
CMOS	Complementary metal–oxide–semiconductor
